# Metabolite, Biochemical, and Dietary Intake Alterations Associated with Lifestyle Interventions in Obese and Overweight Malaysian Women

**DOI:** 10.3390/nu16203501

**Published:** 2024-10-16

**Authors:** Fatin Saparuddin, Mohd Naeem Mohd Nawi, Liyana Ahmad Zamri, Fazliana Mansor, Mohd Fairulnizal Md Noh, Mohd Azahadi Omar, Nur Shahida Abdul Aziz, Norasyikin A. Wahab, Ahmed Mediani, Nor Fadilah Rajab, Razinah Sharif

**Affiliations:** 1 Nutrition, Metabolism and Cardiovascular Research Center, Institute for Medical Research, National Institute of Health, Ministry of Health, Shah Alam 40170, Selangor, Malaysia; 2 Centre of Healthy Ageing and Wellness, Faculty of Health Sciences, Universiti Kebangsaan Malaysia, Kuala Lumpur 50300, Malaysia; 3 Sector for Biostatistic and Data Repository, National Institute of Heath, Ministry of Health, Shah Alam 40170, Selangor, Malaysia; 4 Batu Muda Health Clinic, Ministry of Health, Kuala Lumpur 51100, Malaysia; 5 Department of Medicine, Faculty of Medicine, Universiti Kebangsaan Malaysia, Kuala Lumpur 56000, Malaysia; 6 Institute of Systems Biology, Universiti Kebangsaan Malaysia, Bangi 43600, Selangor, Malaysia; ahmed@ukm.edu.my

**Keywords:** metabolomics, NMR, metabolically healthy obese, lifestyle intervention, obesity

## Abstract

Differences in metabolic regulation among obesity phenotypes, specifically metabolically healthy obese (MHO) and metabolically unhealthy obese (MUO) women, may lead to varied responses to interventions, which could be elucidated through metabolomics. Therefore, this study aims to investigate the differences in metabolite profiles between MHO and MUO women and the changes following a lifestyle intervention. Serum samples from 36 MHO and 34 MUO women who participated in a lifestyle intervention for weight loss were analysed using untargeted proton nuclear magnetic resonance spectroscopy (^1^H NMR) at baseline and 6 months post-intervention. Anthropometric, clinical, and dietary intake parameters were assessed at both time points. Both groups showed differential metabolite profiles at baseline and after six months. Seven metabolites, including trimethylamine-N-oxide (TMAO), arginine, ribose, aspartate, carnitine, choline, and tyrosine, significantly changed between groups post-intervention, which all showed a decreasing pattern in MHO. Significant reductions in body weight and body mass index (BMI) in the MUO correlated with changes in the carnitine and tyrosine levels. In conclusion, metabolite profiles differed significantly between MHO and MUO women before and after a lifestyle intervention. The changes in carnitine and tyrosine levels in MUO were correlated with weight loss, suggesting potential targets for therapeutic intervention.

## 1. Introduction

Globally, 2.5 billion adults are living with overweightness and obesity [1]. In the last 12 years, Malaysia has seen a 10% rise in the prevalence of overweightness and obesity [2]. This trend is not unique to Malaysia, but is evident in many countries globally. The persistent rise in global obesity prevalence remains a significant public health concern worldwide, given its well-established association with an increased risk of developing chronic conditions, such as type 2 diabetes (T2D), hypertension (HPT), and cardiovascular diseases (CVDs) [3,4]. Efforts to combat obesity through various weight loss interventions are widespread. However, the outcomes associated with weight loss intervention vary significantly among individuals.

Variations in the clinical manifestations of obesity could be among the attributes that lead to differences in the responses towards obesity intervention regimens. Weight loss resulting from lifestyle interventions among overweight/obese women categorised as metabolically healthy has been linked to changes in lipid metabolism, activation of sulphation processes, and modulation of microbiota metabolism, potentially indicating a metabolically protective effect [5]. However, gaps persist in understanding disparities in metabolite profiles among obesity phenotypes and the underlying mechanisms that could contribute to differential responses to interventions. A comprehensive investigation is required to clarify the regulatory mechanisms that may vary across different obesity phenotypes.

Metabolomics provides a platform that allows us to capture the dynamic physiological conditions corresponding to current health conditions by analysing low-molecular-weight metabolites present in tissues or biological fluids, such as lipids, amino acids, peptides, organic acids, and carbohydrates [6,7]. This comprehensive metabolite profiling offers valuable insights into the aetiology of obesity and other diseases, facilitating the discovery of potential biomarkers that could enhance our current knowledge of obesity pathophysiology and its related comorbidities.

Hence, this study aims to explore the differences in serum metabolite profiles between women classified as metabolically healthy obese/overweight (MHO) and metabolically unhealthy obese/overweight (MUO) and examine the changes in these metabolite profiles following a six-month lifestyle intervention for weight loss.

## 2. Materials and Methods

### 2.1. Study Design and Study Participants

The present study is a sub-study of an extended lifestyle intervention programme for weight loss, known as “My Body is Fit and Fabulous at Home” (MyBFF@home). The programme involves obese and overweight women, aged 18 to 59 years, with a body mass index (BMI) ≥25.0–≤39.9 kg/m^2^, primarily housewives from low socioeconomic backgrounds residing in low-cost flats in the Klang Valley. Information on the study design, recruitment details, and the intervention programme has been published elsewhere [8,9]. In brief, the previous study divided participants into two groups: obese individuals without comorbidities and obese individuals with comorbidities. Both groups received a 6-month intervention (July–December 2015) consisting of diet control, physical activity (PA), and self-monitoring tools (pedometer, PA, and diet diaries). The PA intervention included a group session held twice a month, featuring 30 to 45 min of brisk walking and 30 min of pillow dumbbell exercises. In addition to the group activity, participants were instructed to perform daily individual exercise sessions, as practised during the group sessions. Participants were provided with an OMRON pedometer (Model HJ321) with a 7-day memory to monitor their daily steps, as well as two pillow dumbbells weighing 300 g each. The 12-step pillow dumbbell exercises were adapted from a Japanese protocol [10]. Additionally, participants maintained a PA diary that included a list of moderate to vigorous housework activities and leisure activities, classified according to the 2011 Compendium of Physical Activities and their respective metabolic equivalent of task (MET) values [11].

**Figure 1:**
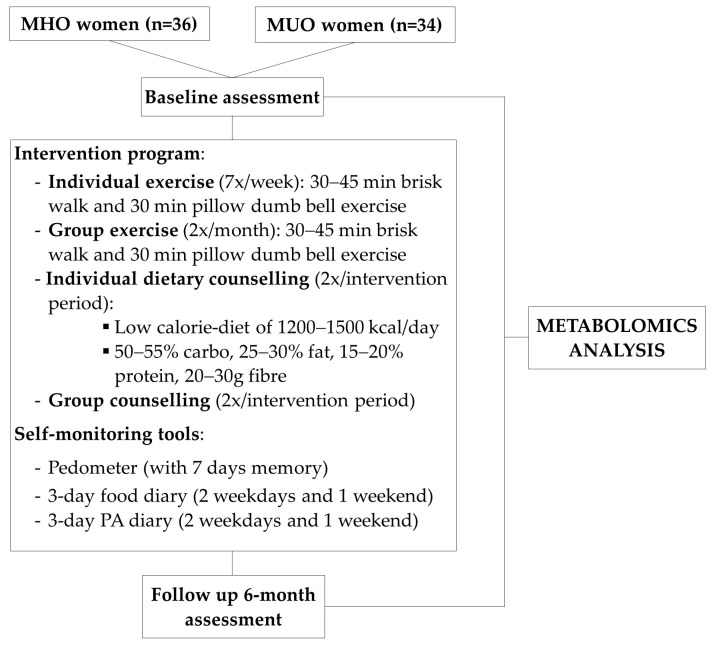
Summary of the lifestyle intervention programme.

For the dietary intervention, participants received individual diet and group counselling sessions with professional dietitians (2 times during the intervention period). The study emphasised low-calorie and low-fat diets. Participants were guided to follow a reduced-calorie-intake diet tailored to their individual energy needs, ranging from 1200 to 1500 kcal per day, with a macronutrient distribution of 50–55% carbohydrates, 25–30% fat, 15–20% protein, and 20–30 g of fibre [12]. Dietary intake was assessed at baseline and at the follow-up visit using a 3-day food diary. Participants were advised to record detailed information on food and beverage types, quantities consumed (measured using household measures or actual weights from labels or packaging), and dietary patterns, including eating out, fast food consumption, and night eating, over two weekdays and one weekend day. Efforts were made to capture foods typically consumed together and specific recipes. Nutrient intake was calculated using Nutritionist Pro TM version 2.4 (First Data Bank, The Hearst Corp, NY, USA). The intervention programme is summarised in Figure 1. Fasting blood samples and anthropometric and clinical measurements were assessed at baseline and after 6 months of intervention. Biochemical profiles were analysed in the laboratory according to methods described previously [13].

This current sub-study randomly selected 70 participants from the previous research, including 36 MHO individuals and 34 MUO individuals. The focus was on those with complete data and serum samples available at the 6-month follow-up. Participants were considered MHO based on the following criteria: HbA1c < 6.5% and systolic or diastolic blood pressure of <140 mm Hg and <90 mm Hg. Meanwhile, MUO participants were categorised according to the following criteria: HbA1c ≥ 6.5%, systolic or diastolic blood pressure of ≥140 mm Hg and ≥90 mm Hg, and self-reported being diagnosed with T2D or HPT [14].

### 2.2. Sample Preparation for Metabolite Profiling

All samples were kept at −80 °C prior to the analysis. Serum samples were thawed on ice to minimise metabolite degradation. Serum samples were vortexed and centrifuged (20,000× *g* × 5 min at 4 °C). Approximately 200 μL of the serum supernatant were mixed with 400 μL of phosphate buffer (KH_2_PO4) at pH 7.4 in deuterium oxide (D_2_O), 0.1% 3-(trimethylsilyl)propionic-2,2,3,3-d_4_ acid sodium salt (TSP) (Merck, Darmstadt, Germany), and 0.1% imidazole (Sigma-Aldrich, St. Louis, MO, USA) in a 1:1 ratio.

### 2.3. NMR Metabolomic Analysis

Untargeted metabolomic approaches were used to analyse the metabolites present in this study. The NMR analysis method was adapted and optimised according to Maulidani et al. [15]. The 1D ^1^H-NMR spectra were collected at a temperature of 26 °C using a 600 MHz Jeol NMR. The combination of PRESAT with the CPMG pulse sequence was used to suppress water signals and wide protein resonances. NMR spectra with a spectral width of 10 ppm were acquired using 128 scans and a 660 s acquisition time. The spectra were processed using the Chenomx NMR suite version 9.0 software (Chenomx Inc., Edmonton, AB, Canada) with the following settings: 0.50 Hz line broadening, autophasing, baseline correction (Whittaker spline), referenced to TSP as an internal standard, and referenced to imidazole as a pH indicator. The spectral band between 0.50 and 10.00 ppm was divided into equal bins using intelligent binning (0.04 ppm). The peak ppm readings were calibrated against the 0 ppm TSP signal. The area of the spectrum associated with residual water and imidazole was eliminated prior to analysis. Subsequently, the corresponding spectra were transformed to a table of common integrals that had a non-negative value for multivariate data analysis (MVDA).

### 2.4. Statistical Analysis

Prior to MVDA, the spectral data were mean-centred and Pareto-scaled to improve normality. Principal component analysis (PCA) and partial least squares discriminant analysis (OPLS-DA) were performed to detect outliers and observe trends and the separation of metabolites between groups using the standard algorithm, as implemented in the SIMCA^®^ software version 17.0.2 (Sartorius, Göttingen, Germany). The validation of the OPLS-DA model was conducted using cross-validated analysis of variance (CV-ANOVA), with the results expressed as *p*-values for the model. Prior to univariate data analysis, the metabolomics data were log-transformed, and metabolites not found in at least 20% of the samples were removed. Statistical analysis was conducted using IBM SPSS Statistics for Windows version 27.0 (Armonk, NY, USA). The normality of continuous data was determined using the Shapiro–Wilk test. Differences in the baseline parameters of the participants between the two groups were analysed using independent t-tests. The generalised estimated equation (GEE) was used to assess significantly changed metabolites between groups (time × group). The analysis was adjusted for sociodemographic characteristics (age, education level, and household income), baseline value of anthropometry parameters (body mass index (BMI) and waist circumference (WC)), fasting plasma glucose (FPG), HbA1c, systolic blood pressure, and the use of medication and baseline value of dietary intake (energy, carbohydrate, protein, total fat, saturated fat, sodium, potassium, and dietary fibre). The Benjamini–Hochberg (B-H) method was applied to correct for multiple testing with the false discovery rate (FDR) set at 5%. Delta values were calculated to assess the extent of changes experienced after the intervention for all participants. These values were derived from the ratio of the sixth-month measurements to the baseline values of significant metabolites, anthropometric measures, blood pressure, and biochemical variables. Pearson correlation analysis was then performed using the delta values to evaluate the relationship between the significant metabolites and anthropometry (weight, BMI, and WC), biochemical (FPG, cholesterol, high-density lipoprotein (HDL), low-density lipoprotein (LDL), and triglyceride (TG)) and clinical data (systolic and diastolic blood pressure).

### 2.5. Pathway Analysis

To explore the metabolic pathways affected by changes in metabolites following the intervention, pathway analysis was conducted in the MetaboAnalyst 6.0 [16] web application, which utilised the databases for *Homo sapiens* from the Kyoto Encyclopaedia of Genes and Genomes (KEGG) and the Human Metabolome Database (HMDB) as the pathway library.

## 3. Results

### 3.1. Baseline Characteristics of Study Participants

The demographic, anthropometric, and clinical characteristics of the study participants (MHO vs. MUO) are presented in Table 1. The significantly different parameters were accounted for in the subsequent statistical analysis.

### 3.2. Anthropometric, Clinical, and Dietary Intake Changes Following 6-Month Intervention

The between-group analysis of the anthropometric and clinical data revealed that body weight and WC showed significant changes post-intervention (*p* < 0.05), as summarised in Table 2. A further within-group analysis revealed that the MUO showed a significant reduction in body weight, BMI, WC, and systolic blood pressure. All dietary intake parameters showed a significant decrease (*p* < 0.05) following the intervention, with the decreased intake evident in both groups.

### 3.3. Multivariate Analysis

An unsupervised PCA was performed using the binned data to observe a potential separation between the MHO and MUO groups at baseline and the sixth month. The baseline PCA score plot (Figure S1) suggests that the metabolomic profiles of the two groups were similar, as indicated by the scattered and random scores on the plot. However, in the sixth month, the score plot in Figure S2 revealed some degree of separation between the groups, despite visible overlapping scores. Further analysis was then performed using the orthogonal projection latent square–discriminant analysis (OPLS-DA) to examine for a more distinct separation of the groups at baseline and the sixth month. In contrast to the corresponding PCA score plot, the baseline OPLS-DA score plot (Figure S3) showed a pattern of separation between MHO and MUO with the goodness of fit, R2 (cum) = 0.336 and predictive ability, Q2 (cum) = 0.207, indicating 33.6% variation in the MHO and MUO metabolomic profiles. Likewise, the sixth-month OPLS-DA score plot (Figure S4) also revealed a distinct separation between MHO and MUO, where the model attributed 62.7% variation in the metabolic profiles of the two groups, with R2 (cum) = 0.627 and Q2 (cum) = 0.140. CV-ANOVA performed on the baseline and sixth-month models resulted in *p* < 0.05, indicating that both models were significant. The anticipated significant clustering of MHO and MUO in the OPLS-DA model was likely attributed to the notable differences in clinical parameters between the two groups. A VIP score of > 1.0 on the VIP plot (Figures S5 and S6) was utilised to pinpoint the regions that discriminated between MHO and MUO metabolic profiles. Regions with negative error bar values were excluded. The corresponding S-plot at baseline and the sixth month, illustrated in Figures S7 and S8, highlighted the discriminating regions (ppm) in red. A detailed examination of the S-plot indicated that the regions with the highest magnitude and reliability in the OPLS-DA model were 3.42 and 3.46 ppm at baseline and 3.42 and 3.74 ppm at the sixth month, denoting the significant role of these regions in differentiating the metabolic profiles between MHO and MUO.

### 3.4. Identification and Relative Quantification of Metabolites

The spectral data were profiled to 39 known metabolites, identified with reference to the Human Metabolome Database (HMDB) using Chenomx, and the relative concentration of each metabolite was obtained by peak fitting with reference to the TSP signal. The identified metabolites are listed in Supplementary Table S1, along with their respective regions. To further understand the differences in metabolite levels between MHO and MUO at the two time points, the relative concentration data of the identified metabolites were exported from Chenomx. The concentration data were log-transformed to improve normality. Subsequently, an independent *t*-test was conducted to evaluate the disparity in metabolite levels between the groups at baseline and the sixth month. The significant metabolites are summarised in Table 3. Three metabolites were observed to be significantly different and higher (*p* < 0.05) in the MUO group than the MHO group at baseline, i.e., glucose, indole-3-acetate, and τ-methylhistidine. Meanwhile, six months after the intervention, 15 metabolites were noted to be significantly different (*p* < 0.05) between the MHO and MUO groups. Fructose was the only metabolite that was significantly lower (*p* = 0.044) in the MUO group. The remaining 14 metabolites, i.e., acetate, arginine, aspartate, betaine, glucose, histidine, isobutyrate, isoleucine, leucine, N-acetylcysteine, phenylacetate, trimethylamine-N-oxide (TMAO), tyrosine, and valine were significantly higher (*p* < 0.05) in the MUO group than the MHO group.

**Figure 2:**
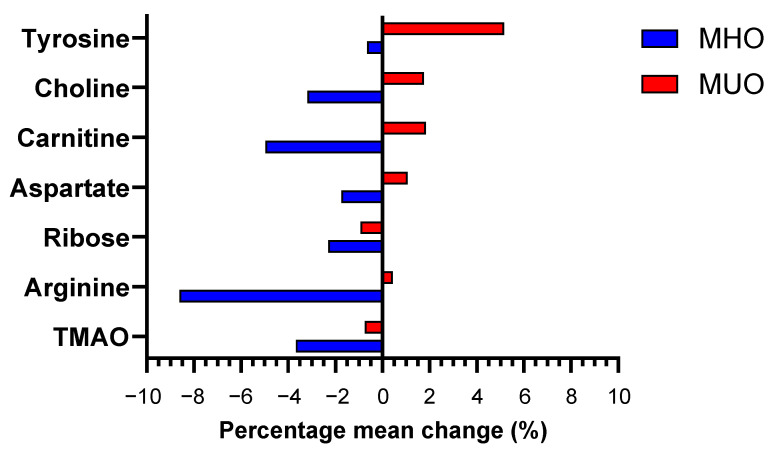
Metabolites that significantly changed between MHO and MUO following intervention.

The changes in metabolite levels from baseline to the sixth month between the groups (time × group effect) were evaluated using GEE analysis. This analysis was adjusted for sociodemographic characteristics (age, education level, and household income), baseline anthropometric parameters (BMI and WC), FPG, HbA1c, systolic blood pressure, medication use, and baseline dietary intake (energy, carbohydrate, protein, total fat, saturated fat, sodium, potassium, and dietary fibre). A Benjamini–Hochberg (B-H) correction was performed to account for FDR, where the metabolites were ranked based on their significant *p*-values. As a result, seven metabolites were found to be significantly different between the groups after six months of intervention: TMAO, arginine, ribose, aspartate, carnitine, choline, and tyrosine. The details of the changes in these seven metabolites are summarised in Table 4 and illustrated in Figure 2.

As illustrated in Figure 2, the changes in metabolite levels in the MHO group were greater than those in the MUO group, except for tyrosine, for which the changes were more prominent in MUO. It is also evident that the changes in all seven metabolites in MHO showed a decreasing pattern after six months of intervention, while in MUO, only two metabolites (TMAO and ribose) showed a decreasing pattern and the other seven metabolites displayed an increasing pattern. The most prominent change was observed in the level of arginine in MHO, which decreased by 8.62% (*p* < 0.05). Meanwhile, tyrosine was the most prominently changed metabolite in the MUO, with a decrease of 5.16% (*p* < 0.05).

### 3.5. Pathway Analysis

**Figure 3:**
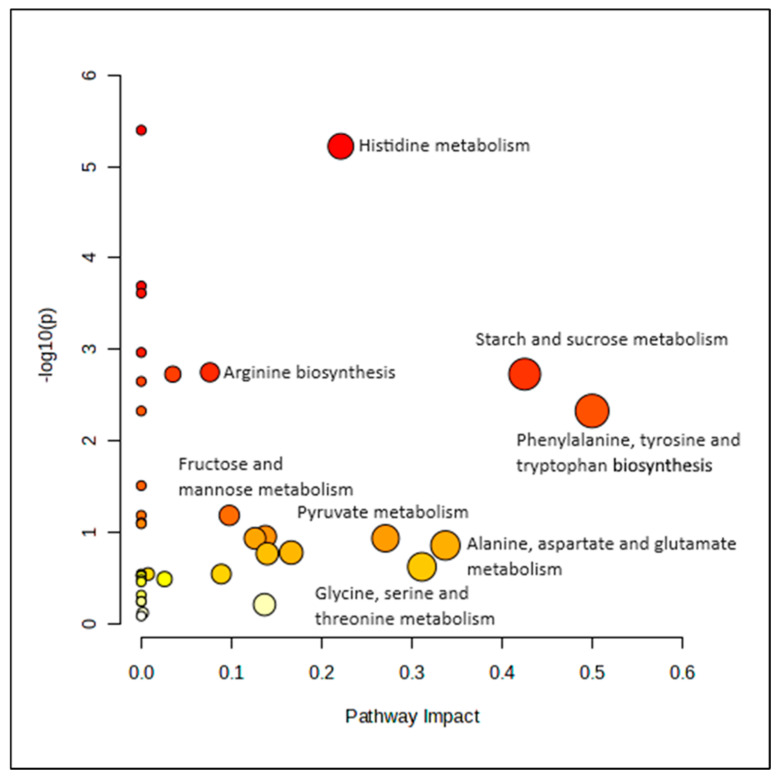
Metabolic pathways affected at sixth month.

Pathway analysis was conducted at two time points using concentration data from 40 metabolites. Initially, no significant pathways were identified at baseline. However, after six months of lifestyle intervention, significant changes in metabolic pathways were observed between MHO and MUO women, as shown in Figure 3. The most impactful pathways were phenylalanine, tyrosine, and tryptophan biosynthesis, with an impact value (piv) of 0.500 and a significant FDR-adjusted *p*-value of 0.016. Additionally, the starch and sucrose metabolism pathways were also significant and impactful, with a piv of 0.425 and a *p*-value of 0.009.

### 3.6. Correlation between Significantly Changed Metabolites with Anthropometry and Clinical Variables

**Figure 4:**
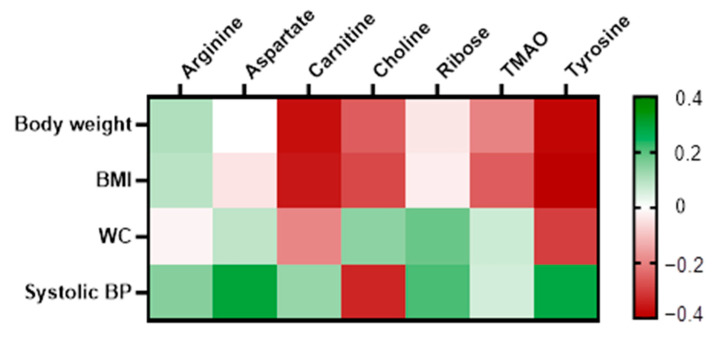
Heatmap of correlation between the significantly changed metabolites with body weight, BMI, WC, and systolic blood pressure in MUO. Correlation determined using Pearson correlation. Green indicates a positive correlation, while red indicates a negative correlation.

Delta values for each variable were computed from the ratio of the sixth-month measurement to its corresponding baseline value. This approach was employed to accommodate the dynamic fluctuations of the variables over the intervention period. The delta values were then utilised to examine the correlation between the metabolites that exhibited significant changes with anthropometric, biochemical, and clinical variables using Pearson correlation analysis. Given the MUO group’s significant reduction in body weight, BMI, WC, and systolic blood pressure, a correlation analysis was conducted to examine the relationship between the significant metabolites and these parameters. The results indicated that carnitine had a significant negative correlation with body weight (r = −0.367, *p* = 0.033) and BMI (r = −0.357, *p* = 0.038). Similarly, tyrosine showed a significant negative correlation with both body weight (r = −0.381, *p* = 0.038) and BMI (r = −0.401, *p* = 0.028), as presented in the heatmap correlation matrix in Figure 4.

**Figure 5:**
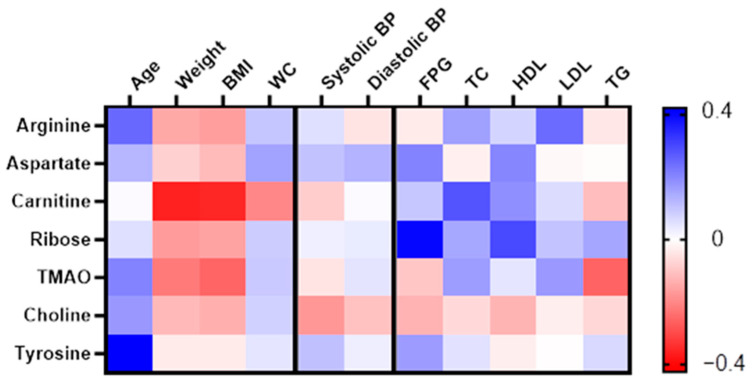
Heatmap of correlation between the significantly changed metabolites with changes in anthropometry, clinical, and biochemical variables. Correlation determined using Pearson correlation analysis in both groups combined. Blue indicates a positive correlation, while red indicates a negative correlation.

A pooled correlation analysis was conducted, irrespective of grouping, to investigate the correlation between the significant metabolites with anthropometry and clinical variables among women with obesity. A heatmap correlation matrix was generated based on the correlation coefficient, r, as shown in Figure 5. No significant correlation was detected with blood pressure parameters. Carnitine displayed a significant negative correlation with changes in weight (r = −0.364, *p* = 0.002) and BMI (r = −0.353, *p* = 0.003) while it exhibited a positive correlation with changes in total cholesterol (TC; r = 0.280, *p* = 0.023). Ribose showed a significant positive correlation with changes in both FPG (r = 0.396, *p* = 0.001) and HDL (r = 0.298, *p* = 0.014). TMAO demonstrated a negative correlation between changes in BMI (r = −0.257, *p* = 0.032) and TG (r = −0.261, *p* = 0.037). Both arginine and tyrosine showed a significant correlation with age (r = 0.248, *p* = 0.040) and (r = 0.421, *p* < 0.001).

## 4. Discussion

In the present study, we observed substantial changes in the metabolomic profiles of MHO and MUO women. However, minimal changes were observed in the anthropometric parameters, and no significant changes were noted in the clinical parameters. Significant age differences were found between the groups, with half of the MUO participants in their 50s, the average age of menopause for Malaysian women [17]. Hormonal changes associated with menopause typically lead to a decrease in basal metabolic rate (BMR) [18], making weight loss more difficult. A weight loss of 5% or more is recommended as clinically meaningful and is often associated with improvements in metabolic health [19,20]. Although the MUO group achieved statistically significant weight loss, it was not clinically meaningful, while the MHO group did not show any significant weight loss. Behavioural and psychological factors might explain this outcome. Older MUO women may have more established routines and habits that support weight loss, like regular meal planning, whereas MHO women in their 40s might have more household responsibilities, hindering consistent compliance with the intervention.

Our study demonstrated that prior to the intervention, the spectral regions of 3.42 and 3.46 ppm were highly predictive of differentiating between MHO and MUO. Following the intervention, regions 3.42 and 3.74 ppm were highly predictive of the separation between MHO and MUO. Based on the spectral data generated and profiled in this study, these regions corresponded to glucose, which was found to be significantly different between the MHO and MUO groups at baseline and six months after intervention. An initial investigation of metabolite differences at baseline identified that three metabolites were significantly different between the MHO and MUO groups. However, after six months of intervention, a greater number of metabolites (15 metabolites) showed significant differences between the groups. Among these were the branched-chain amino acids (BCAAs) leucine, isoleucine, and valine, which have been shown to be associated with insulin resistance, metabolic syndrome, and T2D in various cohorts [21,22,23]. This aligns with our findings, where the levels of BCAAs were significantly higher in MUO women compared with MHO women. The proposed mechanisms linking BCAAs and T2D involve elevated BCAAs levels triggering the activation of the mTOR pathway. This activation increases the production of lipid intermediates, such as diacylglycerol (DAG), which subsequently results in the phosphorylation of insulin receptor substrate (IRS) proteins, impairing downstream insulin signalling [24,25]. The frequent association between BCAAs and obesity and T2D has led to suggestions that MHO and MUO could be characterised based on these metabolites [26].

Further analysis revealed that seven metabolites were significantly changed following the intervention in the MHO and MUO women, i.e., TMAO, arginine, ribose, aspartate, carnitine, choline, and tyrosine. These metabolites displayed a significant decreasing trend in the MHO group. The substantial decrease in circulating choline, carnitine, and TMAO was likely to be attributed to reduced intake of protein-rich foods, such as red meat, which are high in choline and carnitine, during the intervention. TMAO is produced by the gut microbiota from these precursors [27]. Hence, a lower intake of choline- and carnitine-rich foods putatively leads to decreased TMAO levels. This direct relationship was demonstrated previously, showing that the consumption of fish and meat is associated with plasma concentrations of TMAO [28]. Additionally, the marked reduction in arginine levels in MHO could also be linked to the decreased protein intake, as studies have shown that the dietary intake of arginine is significantly associated with the serum and plasma arginine levels, suggesting that a lower intake of arginine-rich foods during the intervention may contribute to this observed decrease [29,30].

Tyrosine is an aromatic amino acid categorised as non-essential, since it can be synthesised in our body from its precursor, phenylalanine. Our study does not only demonstrate that tyrosine levels were markedly elevated in MUO post-intervention, but were also found to be higher in the MUO than MHO at baseline. Similar to BCAAs, elevated levels of tyrosine have been widely linked to insulin resistance and an increased risk of T2D [23,31,32]. This aligns with our findings, as some participants in the MUO group either had been diagnosed with T2D or exhibited elevated HbA1c levels beyond the normal range. Additionally, tyrosine has been linked to ageing [33], which was also evident in this study, as indicated by the positive correlation between tyrosine levels and age.

In this study, the carnitine levels decreased in MHO, but increased in MUO following the intervention, which was accompanied by a significant reduction in body weight and WC in the latter. Additionally, a significant correlation was found between carnitine and both body weight and BMI. A meta-analysis previously revealed that supplementation with L-carnitine significantly reduced body weight [34]. This could pertain to the role of carnitine in fatty acid metabolism. Carnitine is crucial for transporting fatty acids into the mitochondria, where they undergo β-oxidation and are broken down into short- and medium-chain fatty acids to produce energy [35]. The increased energy expenditure through fatty acid metabolism is presumed to be the factor that promoted weight loss in MUO women. Additionally, during periods of reduced calorie intake and weight loss, it is possible that more carnitine was released from the tissues [36]. This could potentially account for the high levels of carnitine observed in MUO individuals, despite their reduced protein intake. The same pattern of changes and correlation with weight loss was also observed with tyrosine. Previous research has shown that weight reduction is associated with decreased tyrosine levels in overweight/obese individuals [37] and those with metabolic syndrome [38]. However, in the present study, tyrosine levels were elevated in the MUO group after the intervention and were significantly correlated with weight reduction. A study on a 3-week weight loss programme also reported significant weight loss with increased tyrosine levels following the intervention. While elevated levels of tyrosine in MUO individuals have often been reported [39,40,41] and are associated with insulin resistance and T2D [23,31,32], the discrepancy in the findings could potentially be attributed to several factors, including differences in the study population, participants’ dietary intake, and the methods used to measure the metabolites.

We conducted a pooled correlation analysis to investigate the association between the significant metabolites and various anthropometric and clinical parameters using the delta data and found that TMAO, arginine, carnitine, and ribose were correlated with lipid parameters, i.e., TG, LDL, TC, and HDL. These metabolites are mostly involved in energy metabolism, lipid metabolism, and vascular function. TMAO was found to be negatively correlated with both BMI and TG. These correlations were also reported in other studies involving overweight individuals, though they found a positive correlation [42,43]. The correlation might be related to the role of the gut microbiota, as it has been shown to also affect changes in TG levels and BMI [44]. As previously mentioned, the circulating levels of TMAO are influenced by the dietary intake of protein-rich food and its biosynthesis by the gut microbiota [27]. In this study, participants across all groups exhibited a significant reduction in protein intake and a corresponding decrease in TMAO levels. This suggests that changes in dietary protein intake could influence circulating TMAO levels by modulating the metabolism of the gut microbiota [45,46]. Another factor that could also influence the circulating levels of TMAO is the activity of the flavin monooxygenase 3 (FMO3) enzyme that may link TMAO to lipid regulation. FMO3 is not only responsible for the conversion of TMA to TMAO in the liver, but it is also involved in the regulation of lipids via the farnesoid X receptor (FXR) and liver X receptor (LXR), resulting in reduced reverse cholesterol transport to the intestine and accumulation of cholesterol [47,48].

In the context of the positive correlation between arginine and LDL, it is plausible that changes in arginine could be a consequence of obesity, rather than the effect of arginine itself on LDL changes, as it has been reported in a meta-analysis that supplementation with arginine did not have any significant impact on LDL [49]. Apart from excessive body weight, participants in the present study exhibited both impaired fasting glucose and hyperlipidaemia. This aligns with the well-documented coexistence of obesity with insulin resistance, metabolic syndrome, inflammation, and oxidative stress [50]. Chronic inflammation and metabolic abnormalities associated with obesity are thought to increase the activity of arginase 1, an enzyme that competes with nitric oxide (NO) synthase for arginine [51,52]. This competition reduces NO production, which is crucial for maintaining healthy vascular function. A decrease in NO leads to increased production of reactive oxygen species (ROS), further promoting oxidative stress and endothelial dysfunction [51,52]. Consequently, this could result in lipid dysregulation, including elevated LDL levels [53,54], as observed in our study. As previously suggested, the significant reduction in carnitine may have been attributed to decreased protein intake during the intervention. Therefore, the observed association between carnitine and TC might also be due to metabolic disturbances associated with obesity. It is worth noting that studies have shown that supplementation with L-carnitine significantly reduces TC levels [55,56], which contradicted our findings.

We also demonstrated a correlation between ribose-FPG and ribose-HDL. Unfortunately, no previous human study has reported such a correlation. While a small amount of ribose can be sourced from dietary intake, our body’s primary source of ribose is synthesised through the pentose phosphate pathway (PPP) [57]. Obesity is known to be associated with elevated activity of glucose-6-phosphate dehydrogenase (G6PD), the rate-limiting enzyme in the pentose phosphate pathway (PPP). The increase in G6PD activity led to enhanced oxidative PPP activity, resulting in higher NADPH production. The surplus NADPH fuelled NADPH oxidase (NOX)-mediated ROS generation, which exacerbated the inflammatory response and induced DNA damage [58,59,60,61,62]. The non-oxidative phase of the PPP produces ribose-5-phosphate (R5P), a crucial building block for nucleotide synthesis [63]. Obesity-induced DNA damage could putatively increase the non-oxidative PPP activity to boost nucleotide synthesis necessary for DNA repair, leading to reduced circulating levels of ribose, as observed in the present study.

This study has several limitations that need to be acknowledged. Firstly, the inherent challenge of accurately monitoring and ensuring adherence to dietary and physical activity interventions. The significant decrease in the reported caloric intake among the participants without corresponding weight loss raises important considerations. Despite our efforts to ensure accurate reporting through multiple methods, including food intake diaries and periodic interviews by professionals, underreporting and inaccuracies remain a concern as self-reported data tend to be influenced by factors such as social desirability and memory recall bias. Another significant limitation of this study is the notable age difference between the groups, with a significant proportion of the MUO women being menopausal. As previously mentioned, hormonal changes associated with menopause typically lead to a reduction in the BMR [18]. This suggests that the differences in metabolic outcomes may not be solely attributable to MHO vs. MUO status, but could also be influenced by the significant age difference and the associated menopausal status. Thirdly, since the inflammatory cytokines associated with obesity were not measured, the association between obesity-related inflammation and the metabolites could not be verified. Additionally, since the samples were obtained from a community-based intervention programme, the research team lacked access to the medical history of participants with comorbidities (T2D and HPT), particularly the duration since their diagnosis. This is a notable limitation, as existing evidence indicates that metabolite levels significantly change as these diseases progress [64]. Consequently, this factor was uncontrollable in the analysis. These limitations highlight the need for cautious interpretation of the findings and suggest that future studies should incorporate more robust methods for dietary assessment, controlling for age and menopausal status, and considering the inclusion of medical history data to enhance the validity and reliability of the results.

## 5. Conclusions

In conclusion, this study highlights distinct metabolomics profiles between MHO and MUO individuals before and after lifestyle intervention for weight loss, with glucose being a key differentiating metabolite. The intervention also led to significant changes in seven metabolites: TMAO, arginine, ribose, aspartate, carnitine, choline, and tyrosine, with a decreasing pattern in these metabolites observed in the MHO group, potentially linked to reduced protein intake, altered lipid metabolism, and gut microbiota modulation. Interestingly, in this study, although the weight loss observed in the MUO group was not clinically significant, the carnitine and tyrosine levels were associated with the observed weight loss. This finding suggests that carnitine and tyrosine could be considered potential therapeutic targets for weight loss, particularly in MUO women. However, a larger-scale, long-term study is warranted to observe the full extent of these effects. Additionally, future studies should consider the potential interactions with other dietary components and the individual variability in response to interventions. Such research could provide valuable insights into personalised nutrition strategies for effective weight management, particularly for MUO women.

## Figures and Tables

**Table 1:** Demographic, anthropometric, and clinical characteristics of the participants at baseline.

	MHO		MUO		*p*
	*n* = 36		*n* = 34		
Age (year), mean ± SD	41.65 ± 8.46		50.01 ± 6.26		<0.001 *
Age group (year), *n*, %					
18–39	12	33.3	3	8.8	0.004 *
40–49	18	50	14	41.2	
≥50	6	16.7	17	50	
Race, *n*, %					
Malay	33	91.7	30	88.2	0.706
Indian	3	8.3	4	11.8	
Level of education, *n*, %					
Primary school	3	8.6	13	39.4	
Secondary school/tertiary education	32	91.4	20	60.6	0.003 *
Household income (RM), *n*, %					
≤1500	12	33.3	23	67.6	
1501–2500	15	41.7	7	20.6	0.016 *
≥2501	9	25	4	11.8	
Family history, *n*, %					
Diabetes	11	31.4	15	44.1	0.326
Hypertension	16	44.4	20	60.6	0.230
Cardiovascular diseases	3	8.3	6	18.2	0.294
Body weight (kg)	71.20 ± 11.39		75.32 ± 10.27		0.117
BMI (kg/m^2^)	29.66 ± 4.05		31.64 ± 3.45		0.031 *
Waist circumference (cm)	91.07 ± 9.11		96.93 ± 7.94		0.006 *
Systolic BP (mmHg)	113.76 ± 11.67		133.06 ± 20.13		<0.001 *
Diastolic BP (mmHg)	73.06 ± 10.11		84.02 ± 12.98		<0.001 *
FPG (mmol/L)	5.19 ± 0.55		6.21 ± 1.63		0.001 *
HbA1c (%)	5.35 ± 0.55		6.05 ± 1.05		0.002 *
TC (mmol/L)	5.44 ± 1.18		5.30 ± 0.89		0.590
HDL-C (mmol/L)	1.31 ± 0.30		1.32 ± 0.22		0.888
LDL-C (mmol/L)	3.95 ± 1.46		3.98 ± 0.87		0.922
Triglyceride (TG) (mmol/L)	1.15 ± 0.56		1.35 ± 0.47		0.129

Continuous variables are presented as mean ± standard deviation (SD) and categorical variables are presented as count (percentage). *p*-values were determined with independent *t*-test for continuous variables and Pearson’s chi-square test for categorical variables. * Significant at *p*-value < 0.05. Abbreviation—MHO: metabolically healthy obese, MUO: metabolically unhealthy obese, kg: kilogram, cm: centimetres, kg/m^2^: kilogram per metre square, BMI: body mass index, BP: blood pressure, FPG: fasting plasma glucose, TC: total cholesterol, HDL-C: high-density lipoprotein–cholesterol, LDL-C: low-density lipoprotein–cholesterol.

**Table 2:** Anthropometry, clinical, and dietary intake changes between MHO and MUO 6 months post-intervention.

Variables	Group	Estimated Marginal Means (95% CI)		Mean Difference (%)	Within Group	Between Group	
		Baseline	6th Month		*p*	Wald Chi-Square	*p*
Body weight (kg)	MHO	69.68 (65.41, 73.94)	69.52 (65.24, 73.80)	−0.156 (−0.23)	0.719	8.125	0.043
	MUO	72.64 (68.79, 76.50)	71.72 (67.93, 75.51)	−0.926 (−1.30)	0.015		
BMI (kg/m^2^)	MHO	30.78 (30.52, 31.03)	30.74 (30.33, 31.16)	−0.034 (−0.11)	0.853	5.464	0.141
	MUO	30.79 (30.54, 31.04)	30.43 (30.06, 30.79)	−0.362 (−1.17)	0.023		
WC (cm)	MHO	90.01 (85.67, 94.35)	92.77 (88.74, 96.81)	2.761 (3.05)	0.483	12.306	0.006
	MUO	93.18 (91.64, 94.73)	90.86 (89.06, 92.67)	−2.322 (−2.48)	<0.001		
Systolic BP (mmHg)	MHO	117.52 (111.38, 123.66)	121.00 (111.45, 127.56)	3.486 (2.91)	0.105	7.321	0.062
	MUO	123.95 (119.64, 128.27)	114.65 (104.69, 124.61)	−9.309 (−7.36)	0.049		
Diastolic BP (mmHg)	MHO	75.24 (69.39, 81.09)	76.57 (69.81, 83.34)	1.333 (1.76)	0.523	4.629	0.201
	MUO	80.43 (75.43, 85.43)	76.68 (68.91, 84.45)	−3.750 (−4.63)	0.226		
Glucose (mmol/L)	MHO	6.44 (5.75, 7.54)	6.51 (5.94, 7.08)	−0.139 (−2.16)	0.679	0.268	0.966
	MUO	6.53 (5.69, 7.37)	6.43 (5.48, 7.37)	−0.101 (−1.61)	0.847		
TC (mmol/L)	MHO	5.91 (4.65, 7.16)	6.02 (5.17, 6.88)	0.113 (1.93)	0.802	0.444	0.931
	MUO	6.14 (5.26, 7.03)	6.21 (5.34, 7.07)	0.064 (1.05)	0.676		
HDL (mmol/L)	MHO	1.64 (1.16, 2.13)	1.51 (1.30, 1.72)	−0.130 (−8.18)	0.518	3.656	0.301
	MUO	1.49 (1.21, 1.77)	1.54 (1.26, 1.82)	0.053 (3.71)	0.089		
LDL (mmol/L)	MHO	4.66 (3.53, 5.78)	4.96 (4.03, 5.89)	0.305 (6.56)	0.378	1.515	0.679
	MUO	4.79 (3.98, 5.61)	4.93 (4.19, 5.67)	0.134 (2.80)	0.461		
TG (mmol/L)	MHO	2.18 (1.59, 2.77)	1.95 (1.57, 2.33)	−0.229 (−10.13)	0.358	3.84	0.279
	MUO	1.70 (1.32, 2.09)	1.77 (1.31, 2.23)	0.062 (3.46)	0.539		
Calorie intake (kcal)	MHO	1559.99 (1308.06, 1811.93)	1148.70 (915.12, 1382.28)	−411.29 (−26.36)	<0.001	17.287	<0.001
	MUO	1315.36 (1105.33, 1525.39)	1082.19 (855.40, 1308.98)	−233.17 (−17.73)	0.014		
Carbohydrate (g)	MHO	201.41 (167.84, 234.97)	152.50 (118.03, 186.98)	−48.90 (−24.28)	0.005	12.353	0.006
	MUO	169.12 (140.16, 198.08)	142.85 (109.04, 176.66)	−26.27 (−15.53)	0.046		
Cholesterol (g)	MHO	224.64 (173.93, 275.34)	139.40 (83.97, 194.84)	−85.23 (−37.94)	0.007	11.117	0.011
	MUO	168.70 (125.87, 211.53)	139.96 (93.60, 186.32)	−28.74 (−17.04)	0.066		
Dietary fibre (g)	MHO	8.13 (5.11, 11.15)	5.39 (2.85, 7.92)	−2.74 (−33.70)	0.011	11.429	0.01
	MUO	6.95 (4.41, 9.48)	4.82 (2.16, 7.48)	−2.13 (−30.65)	0.036		
Potassium (mg)	MHO	1057.04 (836.51, 1277.57)	846.48 (619.61, 1073.35)	−210.56 (−19.92)	0.023	11.097	0.011
	MUO	969.53 (778.77, 1160.29)	773.54 (564.77, 982.31)	−195.99 (−20.21)	0.016		
Protein (g)	MHO	60.37 (49.24, 71.49)	43.84 (32.56, 55.12)	−16.53 (−27.38)	0.003	19.53	<0.001
	MUO	54.29 (44.73, 63.84)	40.25 (30.23, 50.28)	−14.04 (−25.86)	0.001		
Saturated fat (g)	MHO	15.32 (11.65, 19.00)	11.75 (8.09, 15.40)	−3.58 (−23.37)	0.024	15.063	0.002
	MUO	11.93 (9.10, 14.75)	8.57 (5.18, 11.96)	−3.36 (−28.16)	0.009		
Sodium (mg)	MHO	2188.44 (1748.27, 2628.61)	1685.29 (1314.10, 2056.48)	−503.15 (−22.99)	0.021	8.721	0.033
	MUO	1441.14 (1044.33, 1837.95)	1370.37 (907.81, 1832.93)	−70.77 (−4.91)	0.643		
Total fat (g)	MHO	58.76 (46.94, 70.58)	41.61 (30.99, 52.22)	−17.16 (−29.20)	<0.001	16.557	<0.001
	MUO	46.12 (36.83, 55.40)	37.76 (28.56, 46.95)	−8.36 (−18.13)	0.038		

Data presented as estimated marginal mean (95% CI). *p* < 0.05 was considered significant after GEE analysis in which the analysis was adjusted for age, education level, household income, medication use, baseline BMI, WC, FPG, HbA1c, and systolic blood pressure.

**Table 3:** Serum metabolites resolved by ^1^H NMR at baseline and sixth month in MHO and MUO groups.

Metabolites	MHO (*n* = 36)	MUO (*n* = 34)	*p*
Baseline			
Glucose	4.16 ± 0.06	4.25 ± 0.14	0.001
Indole-3-acetate	2.67 ± 0.16	2.75 ± 0.20	0.047
τ-methylhistidine	2.73 ± 0.15	2.82 ± 0.16	0.021
6th month			
Acetate	2.58 ± 0.26	2.71 ± 0.27	0.030
Arginine	2.57 ± 0.31	2.75 ± 0.27	0.014
Aspartate	3.00 ± 0.21	3.15 ± 0.11	<0.001
Betaine	2.73 ± 0.12	2.85 ± 0.16	0.001
Fructose	3.49 ± 0.08	3.44 ± 0.08	0.044
Glucose	4.15 ± 0.08	4.23 ± 0.12	0.002
Histidine	2.77 ± 0.19	2.92 ± 0.12	<0.001
Isobutyrate	2.69 ± 0.12	2.77 ± 0.17	0.025
Isoleucine	2.87 ± 0.19	2.98 ± 0.24	0.031
Leucine	2.67 ± 0.26	2.82 ± 0.35	0.049
N-acetylcysteine	2.63 ± 0.19	2.72 ± 0.18	0.030
Phenylacetate	2.82 ± 0.21	2.94 ± 0.25	0.024
TMAO	2.66 ± 0.12	2.77 ± 0.14	0.001
Tyrosine	3.12 ± 0.19	3.24 ± 0.18	0.014
Valine	2.93 ± 0.17	3.02 ± 0.18	0.037

Values are reported as mean ± SD (µM). Significance is at *p* < 0.05 based on independent *t*-test.

**Table 4:** Summary of the significantly changed metabolites.

Metabolites	Group	Baseline	6th Month	Mean Difference (%)	Within Group	Between Group
					*p*	*p*
TMAO	MHO	2.80 (2.72, 2.88)	2.70 (2.62, 2.77)	−0.103 (−3.68)	<0.001	<0.001
	MUO	2.79 (2.71, 2.87)	2.77 (2.69, 2.85)	−0.021(−0.75)	0.387	
Arginine	MHO	2.89 (2.76, 3.02)	2.64 (2.51, 2.77)	−0.249 (−8.62)	<0.001	0.002
	MUO	2.80 (2.67, 2.94)	2.81 (2.69, 2.94)	0.012 (0.43)	0.835	
Ribose	MHO	3.64 (3.56, 3.71)	3.55 (3.48, 3.62)	−0.084 (−2.31)	0.008	0.002
	MUO	3.67 (3.61, 3.72)	3.63 (3.57, 3.70)	−0.034 (−0.93)	0.079	
Aspartate	MHO	3.09 (2.97, 3.21)	3.04 (2.93, 3.15)	−0.054 (−1.75)	0.152	0.005
	MUO	3.20 (3.11, 3.28)	3.23 (3.15, 3.31)	0.034 (1.06)	0.142	
Carnitine	MHO	2.78 (2.68, 2.89)	2.65 (2.53, 2.77)	−0.138 (−4.96)	0.001	0.005
	MUO	2.66 (2.54, 2.78)	2.71 (2.60, 2.82)	0.049 (1.84)	0.301	
Choline	MHO	2.75 (2.62, 2.88)	2.67 (2.54, 2.79)	−0.088 (−3.20)	0.066	0.006
	MUO	2.55 (2.43, 2.67)	2.60 (2.47, 2.73)	0.045 (1.76)	0.432	
Tyrosine	MHO	2.93 (2.75, 3.10)	2.91 (2.73, 3.09)	−0.019 (−0.65)	0.821	0.008
	MUO	3.08 (3.92, 3.25)	3.24 (3.10, 3.38)	0.159 (5.16)	0.016	

Metabolites listed according to the rank of *p* between groups. Data presented as estimated marginal mean (95% CI) (µM). Significance is at *p* < 0.05 after GEE analysis in which the analysis was adjusted for age, education level, household income, baseline BMI, WC, FPG, HbA1c, systolic blood pressure, medication use, and baseline dietary intake (energy, carbohydrate, protein, total fat, saturated fat, sodium, potassium, and dietary fibre).

## Data Availability

The raw data supporting the conclusions of this article will be made available by the authors on reasonable request. A proposal with a detailed description of study objectives and a statistical analysis plan will be needed for assessment of requests. Additional materials might also be required during the process of assessment. Deidentified participant data will be provided after approval by the investigators.

## Supplementary Materials

The following supporting information can be downloaded at https://www.mdpi.com/article/10.3390/nu16203501/s1, Table S1: List of metabolites identified at baseline and sixth month; Table S2: PCA model fit at baseline and sixth month; Table S3: OPLS-DA model fit at baseline and sixth month; Figure S1: PCA score plot at baseline; Figure S2: PCA score plot at sixth month; Figure S3: OPLS-DA score plot at baseline; Figure S4: OPLS-DA score plot at sixth month; Figure S5: VIP plot at baseline; Figure S6: VIP plot at sixth month; Figure S7: S-plot for baseline model; Figure S8: S-plot for sixth month model.
