# Supplementary material for: Metabolite, Biochemical, and Dietary Intake Alterations Associated with Lifestyle Interventions in Obese and Overweight Malaysian Women
Source: Nutrients. 2024 Oct 16;16(20):3501. doi: 10.3390/nu16203501 (PMC11510420; doi:10.3390/nu16203501)
Supplement: Supplementary file 1 [file nutrients-16-03501-s001.zip › nutrients-3146697-supplementary.pdf]

Supplementary materials

Table S1 List of metabolites identified at baseline and sixth month

Metabolites	Chemical shifts (ppm)
3-hydroxybutyrate	1.20, 2.30, 2.40, 4.10
Acetate	1.90
Alanine	1.50, 3.80
Alloisoleucine	0.90, 1.30, 1.40, 2.00, 3.70
Arginine	1.60, 1.70, 1.90, 3.20, 3.70, 6.70, 7.20
Aspartate	2.70, 2.80, 3.90
Betaine	3.30, 3.90
Butyrate	0.90, 1.60, 2.20
Caprate	0.80, 1.30, 1.50, 2.10
Carnitine	2.40, 2.50, 3.20, 3.40, 4.60
Choline	3.20, 3.50, 4.10
Citrate	2.50, 2.70
Creatine	3.00, 3.90
Cystine	3.20, 3.40, 4.10
Ethylmalonate	0.90, 1.70, 3.00
Fructose	3.50, 3.60, 3.70, 3.80, 3.90, 4.00, 4.10
Glucose	3.20, 3.40, 3.50, 3.70, 3.80, 3.90, 4.60, 5.20
Glutamate	2.00, 2.10, 2.30, 2.40, 3.70
Glutamine	2.10, 2.20, 2.40, 2.50, 3.70, 6.90, 7.60
Glycine	3.60
Histidine	3.10, 3.20, 4.00, 7.10, 7.90
Indole-3-acetate	3.60, 7.20, 7.30, 7.50, 7.60, 10.00
Isobutyrate	1.00, 2.40
Isoleucine	0.90, 1.00, 1.30, 1.50, 2.00, 3.70
Isovalerate	0.90, 2.00
Lactate	1.30, 4.10
Leucine	1.00, 1.70, 1.80, 3.70
Lysine	1.40, 1.50, 1.70, 1.90, 3.00, 3.70
N-acetylcysteine	2.10, 2.90, 4.40, 8.00
N-acetylglycine	2.00, 3.70, 8.00
Phenylacetate	3.50, 7.30, 7.40
Proline	2.00, 2.30, 3.30, 3.40, 4.10
Pyruvate	2.40
Ribose	3.50, 3.60, 3.70, 3.80, 3.90, 4.00, 4.10, 4.20, 4.80, 4.90, 5.20, 5.40
Trimethylamine-N-oxide	3.20
Tyrosine	3.00, 3.20, 3.90, 6.90, 7.20
Valine	1.00, 2.20, 3.60
Valproate	0.90, 1.30, 1.40, 2.20
τ-methylhistidine	3.00, 3.20, 3.70, 3.90, 7.00, 7.70

Table S2 PCA model fit at baseline and sixth month

PCA Model	Goodness of fit (R^2)	Goodness of prediction (Q^2)
Baseline	0.609	0.525
Sixth month	0.626	0.473

Table S3 OPLS-DA model fit at baseline and sixth month

OPLS-DA Model	Goodness of fit (R^2)	Goodness of prediction (Q^2)	CV ANOVA
Baseline	0.336	0.207	0.006
Sixth month	0.627	0.140	0.045

PCA score plot at baseline (Figure S1) and sixth month (Figure S2)

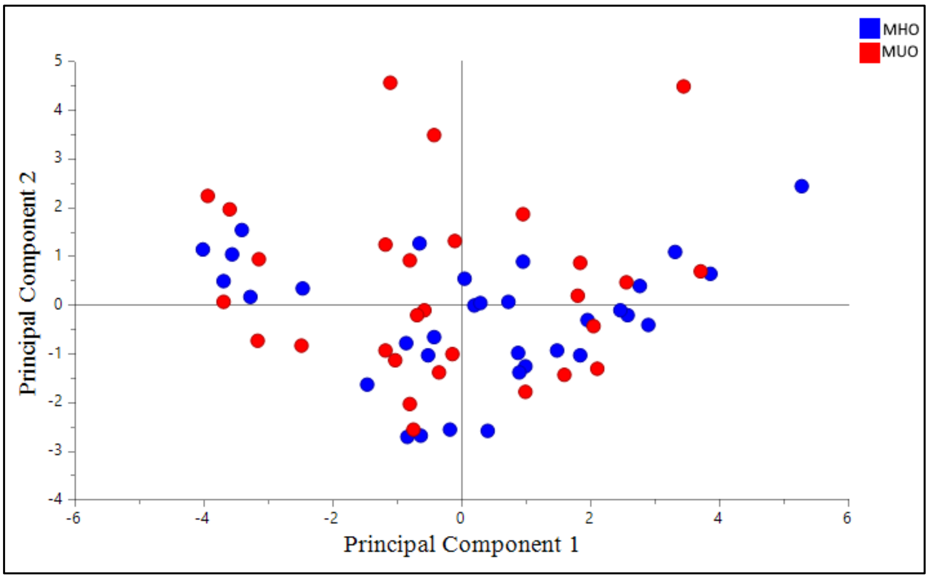

Figure S1 PCA score plot at baseline

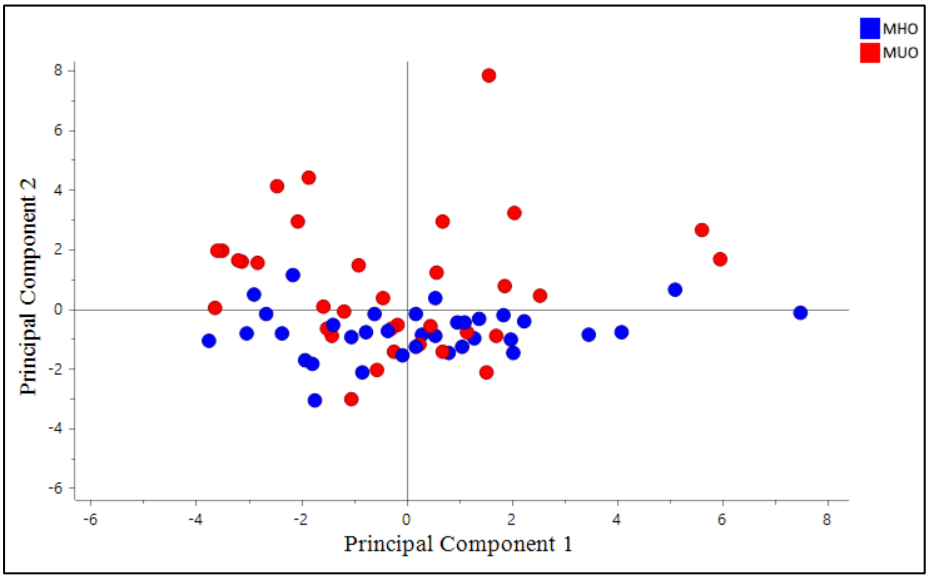

Figure S2 PCA score plot at sixth month

OPLS-DA score plot at baseline (Figure S3) and sixth month (Figure S4)

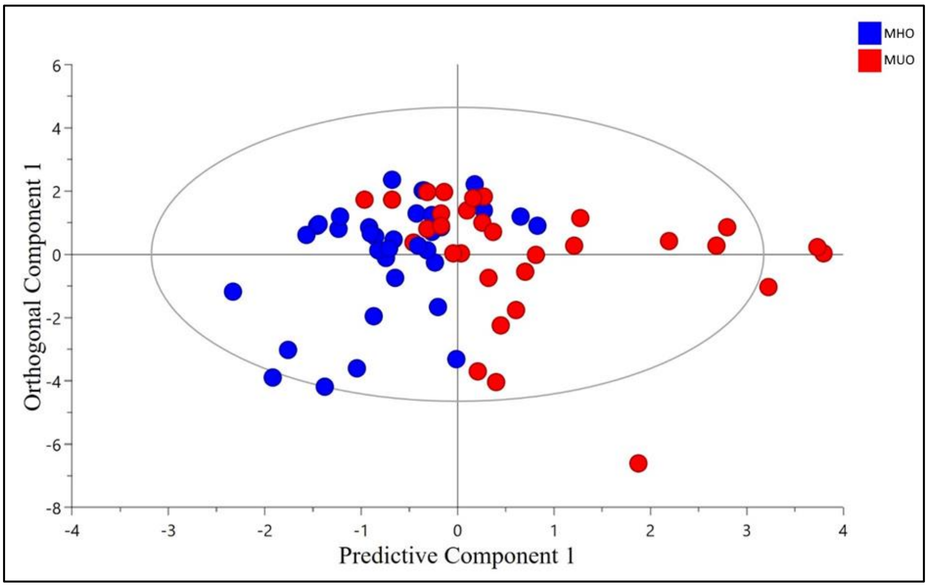

Figure S3 OPLS-DA score plot at baseline

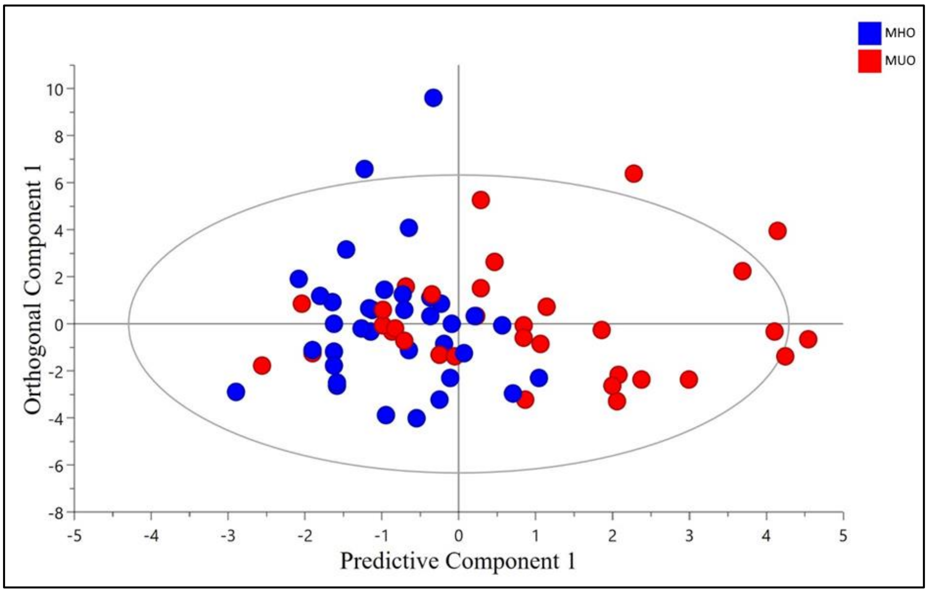

Figure S4 OPLS-DA score plot at sixth month

VIP plot for baseline (Figure S5) and sixth month model (Figure S6)

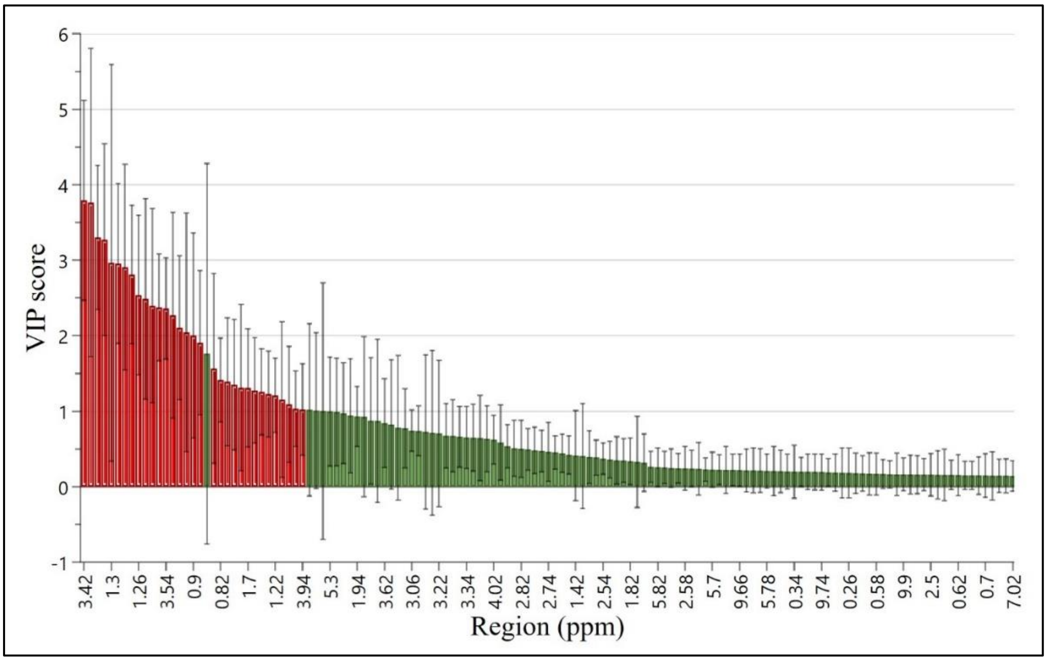

Figure S5 VIP plot at baseline.

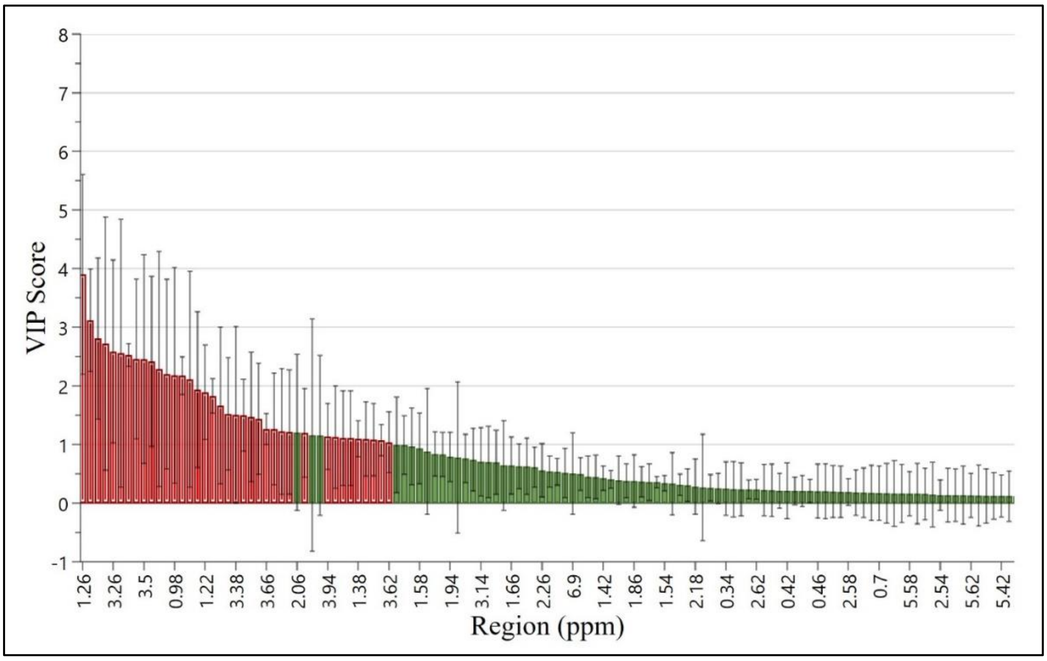

Figure S6 VIP plot at sixth month.

S-plot for baseline (Figure S7) and sixth month model (Figure S8)

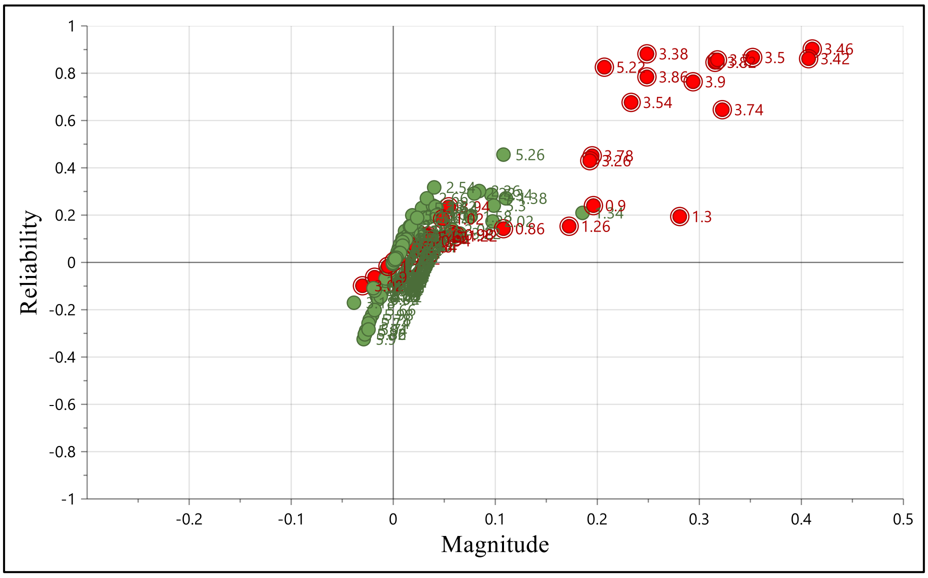

Figure S7 Corresponding S-plot for baseline model. Regions (ppm) with VIP score > 1.0 highlighted in red in the S-plot were responsible for the separation of the MHO and MHO group.

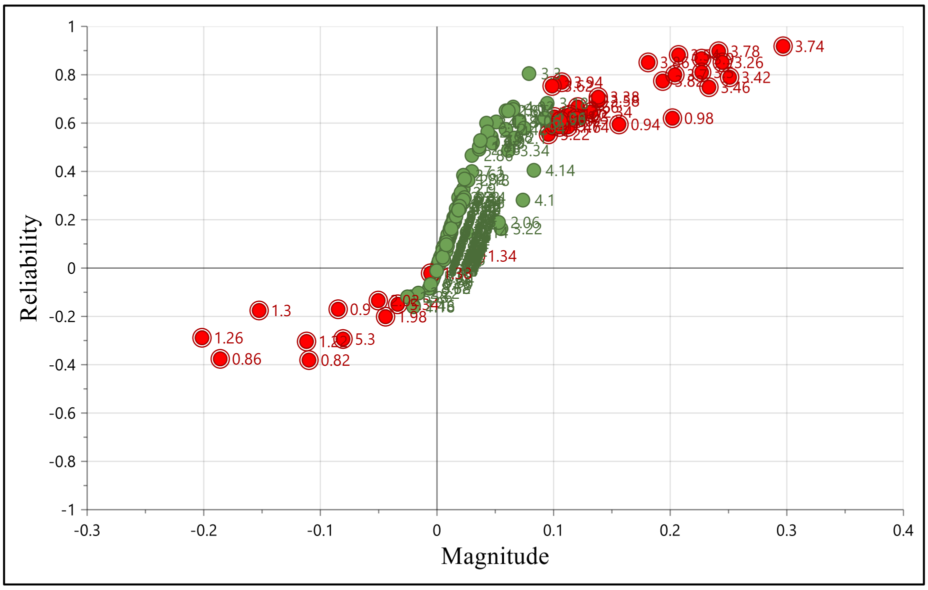

Figure S8 Corresponding S-plot for sixth month model. Regions (ppm) with VIP score > 1.0 highlighted in red in the S-plot were responsible for the separation of the MHO and MHO group.
